# Psychiatric Comorbidity Does Not Enhance Prescription Opioid Use in Inflammatory Bowel Disease as It Does in the General Population

**DOI:** 10.1093/ibd/izae188

**Published:** 2024-09-03

**Authors:** Charles N Bernstein, John D Fisk, Randy Walld, James M Bolton, Jitender Sareen, Scott B Patten, Alexander Singer, Lisa M Lix, Carol A Hitchon, Renée El-Gabalawy, Alan Katz, Lesley A Graff, Ruth Ann Marrie, for the CIHR Team in Defining the Burden and Managing the Effects of Psychiatric Comorbidity in Chronic Immunoinflammatory Disease, Ruth Ann Marrie, James M Bolton, Jitender Sareen, Scott B Patten, Alexander Singer, Lisa M Lix, Carol A Hitchon, Renée El-Gabalawy, Alan Katz, John D Fisk, Charles N Bernstein, Lesley Graff, Lindsay Berrigan, Ryan Zarychanski, Christine Peschken, James Marriott, Kaarina Kowalec, Lindsay Berrigan

**Affiliations:** Rady Faculty of Health Sciences, Department of Internal Medicine, Max Rady College of Medicine, University of Manitoba, Winnipeg, Manitoba, Canada; IBD Clinical and Research Centre, University of Manitoba, Winnipeg, Manitoba, Canada; Nova Scotia Health and the Departments of Psychiatry, Psychology & Neuroscience, and Medicine, Dalhousie University, Halifax, Nova Scotia, Canada; Rady Faculty of Health Sciences, Manitoba Centre for Health Policy, Max Rady College of Medicine, University of Manitoba, Winnipeg, Manitoba, Canada; Rady Faculty of Health Sciences, Department of Psychiatry, Max Rady College of Medicine, University of Manitoba, Winnipeg, Manitoba, Canada; Rady Faculty of Health Sciences, Department of Psychiatry, Max Rady College of Medicine, University of Manitoba, Winnipeg, Manitoba, Canada; Department of Community Health Sciences, Cumming School of Medicine, University of Calgary, Calgary, Alberta, Canada; Rady Faculty of Health Sciences, Department of Family Medicine, Max Rady College of Medicine, University of Manitoba, Winnipeg, Manitoba, Canada; Rady Faculty of Health Sciences, Department of Community Health Sciences, Max Rady College of Medicine, University of Manitoba, Winnipeg, Manitoba, Canada; Rady Faculty of Health Sciences, Department of Internal Medicine, Max Rady College of Medicine, University of Manitoba, Winnipeg, Manitoba, Canada; Rady Faculty of Health Sciences, Department of Clinical Health Psychology, Max Rady College of Medicine, University of Manitoba, Winnipeg, Manitoba, Canada; Rady Faculty of Health Sciences, Department of Anesthesiology, Perioperative Medicine and Pain, Max Rady College of Medicine, University of Manitoba, Winnipeg, Manitoba, Canada; Rady Faculty of Health Sciences, Manitoba Centre for Health Policy, Max Rady College of Medicine, University of Manitoba, Winnipeg, Manitoba, Canada; Rady Faculty of Health Sciences, Department of Family Medicine, Max Rady College of Medicine, University of Manitoba, Winnipeg, Manitoba, Canada; Rady Faculty of Health Sciences, Department of Community Health Sciences, Max Rady College of Medicine, University of Manitoba, Winnipeg, Manitoba, Canada; IBD Clinical and Research Centre, University of Manitoba, Winnipeg, Manitoba, Canada; Rady Faculty of Health Sciences, Department of Clinical Health Psychology, Max Rady College of Medicine, University of Manitoba, Winnipeg, Manitoba, Canada; Rady Faculty of Health Sciences, Department of Internal Medicine, Max Rady College of Medicine, University of Manitoba, Winnipeg, Manitoba, Canada; IBD Clinical and Research Centre, University of Manitoba, Winnipeg, Manitoba, Canada; Rady Faculty of Health Sciences, Department of Community Health Sciences, Max Rady College of Medicine, University of Manitoba, Winnipeg, Manitoba, Canada

**Keywords:** opioids, psychiatric comorbidity, inflammatory bowel disease, population-based

## Abstract

**Introduction:**

Little is known about patterns of opioid prescribing in inflammatory bowel disease (IBD), but pain is common in persons with IBD. We estimated the incidence and prevalence of opioid use in adults with IBD and an unaffected reference cohort and assessed factors that modified opioid use.

**Methods:**

Using population-based health administrative data from Manitoba, Canada, we identified 5233 persons with incident IBD and 26 150 persons without IBD matched 5:1 on sex, birth year, and region from 1997 to 2016. New and prevalent opioid prescription dispensations were quantified, and patterns related to duration of use were identified. Generalized linear models were used to test the association between IBD, psychiatric comorbidity, and opioid use adjusting for sociodemographic characteristics, physical comorbidities, and healthcare use.

**Results:**

Opioids were dispensed to 27% of persons with IBD and to 12.9% of the unaffected reference cohort. The unadjusted crude incidence per 1000 person-years of opioid use was nearly twice as high for the IBD cohort (88.63; 95% CI, 82.73-91.99) vs the reference cohort (45.02; 95% CI, 43.49-45.82; relative risk 1.97; 95% CI, 1.86-2.08). The incidence rate per 1000 person-years was highest in those 18-44 years at diagnosis (98.01; 95% CI, 91.45-104.57). The relative increase in opioid use by persons with IBD compared to reference cohort was lower among persons with psychiatric comorbidity relative to the increased opioid use among persons with IBD and reference cohort without psychiatric comorbidity.

**Discussion:**

The use of opioids is more common in people with IBD than in people without IBD. This does not appear to be driven by psychiatric comorbidity.

**Key Messages**

- What is known?

• Both pain and opioid use are common in inflammatory bowel disease (IBD), but little is known about patterns of opioid prescribing in IBD, and to what extent psychiatric comorbidities impact opioid use.

- What is new here?

• While 27% of persons with IBD were dispensed opioids and the incidence rate was twice as high for the IBD cohort compared to a matched unaffected reference cohort; psychiatric comorbidity had less impact on opioid use in IBD than in an unaffected reference cohort.

## Introduction

Inflammatory bowel disease (IBD) includes Crohn’s disease (CD) and ulcerative colitis (UC). Abdominal pain and joint pain are common manifestations even among persons in remission.^1,2^ Psychiatric comorbidity is also highly prevalent in persons with IBD at approximately twice the rate of persons without IBD.^3^ Underlying depression can intensify pain.^4^ The most effective approach to treating pain occurring in the context of IBD is to target the underlying causes where possible, such as the active inflammatory luminal, perineal, or joint disease. Proton pump inhibitors have been used for certain types of pain.^5^ Nonsteroid anti-inflammatory drugs (NSAIDs) are used to treat arthralgias, although there is debate over their potential to detrimentally affect the course of IBD.^6^ Opioids have also been used as part of the arsenal for IBD treatment, targeting pain or to reduce bowel movement frequency.^7,8^

Psychiatric comorbidity has been associated with opioid-induced hyperalgesia and opioid misuse in the setting of low back pain.^9^ In a systematic review of opioid use, psychiatric comorbidity was associated with long-term opioid use.^10^ Further, the concomitant use of selective serotonin reuptake inhibitors (SSRIs) that inhibit cytochrome-P450 2D6 (CYP2D6) enzyme may increase the risk of opioid overdose.^11^ In 2014, we reported that 5% of persons with IBD use opioids to excess and were 4 times more likely to become heavy opioid users than matched controls.^12^ Heavy opioid use by persons with IBD was a strong predictor of mortality.

Given concerns about the potential harms related to opioid use^13^ and limited data supporting their utility for pain management specifically in IBD, we characterized their use in IBD overall, and in relation to psychiatric comorbidity. We estimated the incidence, prevalence, and patterns (frequency and duration) of opioid prescription in the adult IBD population and a matched reference cohort without IBD. Further, we aimed to determine if psychiatric comorbidity was a principal driver of opioid use in persons with IBD compared with a reference cohort.

## Methods

### Setting and Data Sources

This matched retrospective cohort study was conducted in Manitoba, a central Canadian province with a population of nearly 1.4 million. Every Manitoba resident has a unique personal health identification number (PHIN), and services are provided through a publicly funded universal healthcare system. All healthcare contacts for outpatient physician visits, hospitalizations, and medication dispensing are connected to the PHIN for each individual. The health administration databases accessed for the study included the population registry (dates of birth, death, and health care coverage; sex; and postal code which provided region of residence), medical services/physician claims, the hospital Discharge Abstract Database and Drug Program Information Network (DPIN). Medical services and hospitalization data have been available since 1984 whereas DPIN data have been available since 1995.

The University of Manitoba Health Research Ethics Board and Manitoba’s Health Information Privacy Committee approved this study (Research Ethics Board number: H2014:201).

### Study Populations

All Manitobans with IBD over the period 1984-2016 were identified by applying a validated case definition that required at least 5 healthcare encounters (hospitalizations or physician visits) for IBD, or at least 3 IBD encounters if registered for less than 2 years,^14^ based on diagnostic codes for IBD. The index date for each person with IBD was based on the earliest CD or UC claim. CD was identified by International Classification of Disease (ICD)-9 code 555 or ICD-10 code K51, and UC by ICD-9 code 556 and ICD-10 code K52. We created a matched general population cohort with 5:1 matching to the IBD cohort based on sex, year of birth (±5 years), and forward sortation area (first 3 digits of postal code). The reference cohort excluded individuals with diagnosis codes for IBD, as well as multiple sclerosis and rheumatoid arthritis because we conducted a series of studies that required a common control group to support comparisons across diseases. The index date for each person in the reference cohort was the index date of their matched case. Working from these prevalent cohorts, we focused on incident IBD cases and the matched reference cohort with an index date of 1997 or later. This allowed for at least a 1-year run-in period as DPIN data were available from 1995. We also excluded individuals in the IBD and persons in the reference cohorts with cancer (based on ≥1 ICD-9 140-208 or ICD-10-CA C00-C97 code) or in palliative care (based on hospital ICD-9/ICD-10-CA V66.7/Z51.5) since these could be indications for opioid prescription.^15^ Individuals were censored when they were first diagnosed with cancer or entered palliative care, on death, or on leaving the province.

### Psychiatric Comorbidity

We focused on any mood or anxiety disorder because of the availability of a validated case definition, and because these are the most common psychiatric disorders in the general and IBD populations. We applied validated case definitions to identify persons with any diagnosed mood or anxiety disorder (including at least one of depression, anxiety, or bipolar disorders).^16^ The ICD-9 codes and ICD-10 codes used to define psychiatric comorbidity are in Supplementary Table S1. The case definition did not include drug exposures, since some antidepressants are used in the management of chronic pain. The date of the first claim for each condition was designated as the diagnosis date. If there were at least 2 physician claims or 1 hospital claim with a diagnosis code for the mood/anxiety disorder in any subsequent year after meeting the case definition the person was deemed to be an “active” psychiatric comorbidity case in that year; for hospital claims, the mood/anxiety disorder had to be the most responsible diagnosis.^17^

### Opioid Use

Opiates are chemical compounds extracted or refined from natural plant matter (ie, morphine or codeine), while opioids are synthesized chemical compounds (ie, hydrocodone, oxycodone, fentanyl). In this study, the term opioid is used to refer to all opiates and opioids. Opioids were identified based on DINs recorded in the DPIN linked to the World Health Organization’s Anatomical Therapeutic Chemical (ATC) Classification System (N02AA03, N02AA01, N02AA05, N02AB03, N02AB02).^18^ Incident users of opioids were defined as those persons with no dispensation for any opioid for at least 1 year before initial dispensation. Prevalent opioid users were defined as persons with at least 1 opioid dispensation in the year of interest.

Time from the first dispensation of an opioid to discontinuation of therapy (the latter defined as a gap of at least 90 days between dispensations) was measured, as were patterns of use based on dispensation dates and number of days supplied at dispensation.^19^ These analyses were restricted to cohort members who had at least 5 years of follow-up post-index date. We report on short-term use (less than 3 months) and chronic use (at least 3 months) within that 5-year period. The 3-month or more time frame to identify chronic use was selected because it has been the most common definition of long-term opioid use in the literature.^9^ In addition to looking at (i) single dispensation, to further examine chronic use, we report the percentage (95% confidence interval [95% CI]) for (ii) continuous use for at least 3 months; (iii) cumulative use for at least 3 months in any 1-year period; (iv) continuous use for at least 6 months; and (v) cumulative use of at least 6 months in a 1-year period. Because of the 5-year period, discontinuation of opioids could be followed by re-initiation of therapy, and the total percentages could exceed 100%.

Since other medications may also be used for chronic pain management, we report the frequency of use of at least 1 prescription for each of nonsteroidal anti-inflammatory drugs (NSAIDs), cannabinoids (A04AD11, A04AD10, N02BG10), tricyclic antidepressants (TCA [N06AA]), and selective serotonin and norepinephrine reuptake inhibitors (SSRIs [N06AB], SNRIs [N06AB]) in the year before the first opioid dispensation.

### Covariates

Demographic data, health service use, and clinical characteristics were covariates used in the analysis. As described previously and consistent with our prior work,^20^ sociodemographic covariates included sex (male as reference group), current age (18-44 [reference group], 45-64, ≥65), region of residence (urban, rural as reference), and socioeconomic status (SES, continuous). We calculated the Socioeconomic Factor Index version 2 (SEFI-2) which integrates average household income, high school education rates, unemployment rates, and percent of single-parent households into a single score as a measure of SES.^21^ Scores greater than 0 indicate lower SES, whereas scores less than 0 indicate more favorable socioeconomic conditions. Health service use covariates included annual number of physician visits (0-3 [reference], 4-7, ≥7) and annual number of classes (types) of prescription medications dispensed, at the fourth level of the ATC system (eg, by chemical subgroup) after excluding opioids (0-1, 2-3, ≥4). Clinical characteristics included index year (continuous), disease duration from the index date (continuous), and number of physical comorbidities (0 [reference group], 1, ≥2). We used the John Hopkins Adjusted Clinical Group System Aggregated Diagnosis Groups (ADGs) to describe health comorbidities,^22,23^ specifically the chronic (not time limited) major physical ADGs. For analyses of incident opioid use, we included physical comorbidity, number of physician visits, and number of medication classes used in the year before the first dispensation as covariates. For analyses of prevalent opioid use, we updated these variables annually. Analyses were undertaken for IBD, as well as for CD and for UC, separately.

### Analysis

Descriptive statistics were used to characterize the IBD and the reference cohorts including frequency (percent), mean (standard deviation), and median (interquartile range [IQR]). We estimated the crude incidence rate (new users) and prevalence of any opioid use, overall as well as stratified by sex, and age (18-44, 45-64, ≥65 years) for each year in the study period. Incidence rates and prevalence estimates were age- and sex-standardized to the 2010 Canadian population from the Statistics Canada census; 95% CI was based on a negative binomial distribution. We compared incidence rates and prevalence between the IBD and the reference cohorts using rate ratios and 95% CI. We used generalized linear models to test the association of disease group (IBD vs reference cohort), mood and anxiety disorders, and their interaction with opioid use. These models used a binomial distribution and included the natural log of person-time as the model offset; for the prevalence analyses, we used generalized estimating equations with an exchangeable correlation structure. Covariates were those described above.

Statistical analyses were conducted using SAS V9.4 (SAS Institute Inc., Cary, NC).

## Results

The incident IBD cohort included 5233 persons and the reference cohort included 26 150 persons. The cohorts were similar with respect to age, sex, and region of residence. Slightly more than half of IBD participants were female and two-thirds resided in urban centers (Table 1). In the IBD cohort, 72.5% were followed to study completion. Reasons for the truncation of IBD cohort participants were cancer diagnosis (14.9%), death (3.7%), moving out of province (8.7%), and palliative care (0.2%) (Supplementary Table S2).

**Table 1.** IBD incident cohorts: 1997-2017—distribution of demographics.

Variable	
Sex (female)	2815 (53.8%)
Residence (urban)	3433 (65.6%)
Index year	
1997-2001	1335 (25.5%)
2002-2006	1207 (23.1%)
2007-2011	1207 (23.1%)
2012-2017	1484 (28.4%)
SEFI mean (SD)	-0.22 (0.88)
SEFI median	-0.23 (-0.75, 0.26)
Mean age at index (SD), years	41.7 (16.5)
Mean years of follow-up (SD), years	8.57 (5.91)
Physician visits number in year prior to index mean (SD)	6.36 (6.45)
Use of NSAIDs in the year prior to opioid use^a^	197 (13.1%)
Use of cannabinoids in the year prior to opioid use^a^	
Use of TCAs in the year prior to opioid use*	55 (3.9%)
Use of SSRI in the year prior to opioid use	101 (7.1%)
Use of SNRI in the year prior to opioid use	30 (2.1%)

Abbreviations: NSAID, nonsteroidal anti-inflammatory drugs; SD, standard deviation; SEFI, socioeconomic factor index; SNRI, selective norepinephrine reuptake inhibitors; SSRI, selective serotonin reuptake inhibitors and norepinephrine reuptake inhibitors; TCA, tricyclic antidepressants.

^a^Refers to use of at least 1 prescription of the listed medications in the year prior to the first opioid dispensation: NSAID, cannabinoids, TCA, SSRI, and SNRI.

In the IBD cohort, 1420 persons were ever dispensed opioids (27.1%) versus 5693 (21.8%) persons from the reference cohort. In the year before the initial opioid dispensation, 55 of 1420 persons (3.9%) with IBD had at least 1 prescription dispensation for a TCA, 101 (7.1%) for an SSRI, 30 (2.1%) for an SNRI, and 197 (13.1%) for an NSAID. The proportion of persons using TCA, SSRI, and SNRI were all significantly greater in IBD relative to the reference cohort, whereas the use of NSAIDs was lower in IBD cases (Supplementary Table S3). Cannabinoids were prescribed in less than 6 persons with IBD so that result must be suppressed.

### Incidence of Opioid Use

In the IBD cohort, the unadjusted crude incidence per 1000 person-years of opioid use over the study period was 88.63 (95% CI, 82.73, 91.99). This was similar to the age and sex- standardized incidence rate per 1000 person-years of 87.24 (95% CI, 82.73, 91.99). The average age-standardized incidence rate was highest in those 18-44 years at diagnosis per 1000 person-years (98.01; 95% CI, 91.45, 104.57) (Supplementary Figure S1). On unadjusted analyses, the rate of opioid use was nearly 2-fold higher among participants with IBD than a reference cohort (RR = 1.97; 95% CI, 1.86-2.08). The relative risk (RR) of approximately 2-fold for cases versus the reference cohort was similar across the age span (*P* = .66) and by sex (*P* = .66) (Table 2).

**Table 2.** Opioid incidence rates (per 1000) and rate ratios: IBD incident cohort 1997-2017 by age category and by sex compared to the reference cohort and rate ratio (RR) with 95% confidence interval (CI).

	Total IBD, “*n*”	Opioid users, “*n*”	Case rate (95% CI)	Reference cohort, “*n*”	Opioid users, “*n*”	Reference cohort rate	Rate ratio (95% CI)
Age category							
18-44 years	8754	858	98.01 (91.45, 104.57)	63 201	3182	50.35 (48.60, 52.10)	1.95 (1.81, 2.09)
45-64 years	5321	404	75.92 (68.52, 83.32)	44 749	1789	39.98 (38.13, 41.83)	1.90 (1.71, 2.11)
65+ years	1946	158	81.20 (68.54, 93.87)	18 500	722	39.03 (36.18, 41.87)	2.08 (1.76, 2.46)
Sex category							
Males	7879	692	87.83 (81.29, 94.38)	58 722	2654	45.20 (43.48, 46.92)	1.94 (1.79, 2.11)
Females	8143	728	89.40 (82.91, 95.90)	67 728	3039	44.87 (43.28, 46.47)	1.99 (1.84, 2.15)

Abbreviation: IBD, inflammatory bowel disease.

16 022 cases by male and female and 16 021 by age.

On multivariable analysis, the relative rate of opioid use among participants with IBD with psychiatric comorbidity (RR 1.21; 95% CI, 0.99-1.48) was lower than among participants with IBD without psychiatric comorbidity (RR 1.39; 95% CI, 1.29-1.50). Among persons with IBD, psychiatric comorbidity was not associated with a significantly increased incidence of opioid use in the IBD (RR 0.93; 95% CI, 00.78, 1.12) or reference cohorts (RR 1.07; 95% CI, 0.97-1.19); no interaction was observed for the combination of having IBD and a psychiatric comorbidity on opioid use.

Older age, being female, longer disease duration, and later year of diagnosis were all associated with lower rates of incident opioid use (Table 3). Having more medical comorbidities, being on more prescription drug classes, having more than 3 annual physician visits, and being in the poorest socioeconomic quintile were all associated with increased incidence of opioid use. However, these outcomes were similar for CD and for UC when they were analyzed separately (see Supplementary Tables S4 and S5).

**Table 3.** Factors associated with incident opioid use (incidence rate ratio, 95% confidence interval, CI) Effect estimates for adjustment covariates for the incident IBD cohort.

Variable	Effect estimate	95% CI
Age 45-64 vs 18-44	0.80	(0.75, 0.84)
Age 65+ vs 18-44	0.69	(0.64, 0.75)
Female vs male	0.92	(0.87, 0.96)
Urban vs rural	1.00	(0.95, 1.05)
ADG 1 vs 0	1.17	(1.09, 1.25)
ADG 2+ vs 0	1.34	(1.13, 1.58)
ATC4 drug classes 2-3 vs 0-1	1.20	(1.13, 1.28)
ATC4 drug classes 4+ vs 0-1	1.31	(1.21, 1.41)
Prior IBD-specific procedure	1.04	(0.84, 1.28)
Prior physician visits: 4-7 vs 0-3	1.29	(1.21, 1.38)
Prior physician visits: 8 or more vs 0-3	1.37	(1.26, 1.48)
SEFI: Poorest vs. Richest quartile	1.11	(1.03, 1.19)
IBD disease duration among cases (1 extra year)	0.88	(0.87, 0.89)
IBD diagnosis year	0.93	(0.93, 0.94)
CASE*PSYCH interaction term	0.87	(0.71, 1.07)

Abbreviations: ADG, John Hopkins Adjusted Clinical Group System Aggregated Diagnosis Groups; ATC4, Anatomical Therapeutic Chemical (ATC) Classification System 4; SEFI, Socioeconomic Factor Index; CASE*PSYCH interaction term, IBD case vs, reference cohort, with and without anxiety and/or mood disorders.

For individuals with CD and without psychiatric comorbidity, the unadjusted crude incidence per 1000 person-years of opioid use was 101.8 (95% CI, 77.7, 133.3) and for those with psychiatric morbidity, it was 108.2 (95% CI, 99.9, 117.2). The rate of incident opioid use did not differ between persons with CD with and without psychiatric comorbidity (RR 0.94; 95% CI, 0.71, 1.25). Among persons with psychiatric comorbidity, those with CD had a 73% higher rate of incident opioid use compared to a matched reference cohort (RR 1.73; 95% CI, 1.28, 2.34). However, among persons without psychiatric comorbidity, the relative increase in the rate of incident opioid use among those with CD compared to a matched reference cohort was much higher (RR 2.36; 95% CI, 2.36, 2.58).

For UC without psychiatric comorbidity, the unadjusted crude incidence per 1000 person-years of opioid use was 76.2 (95% CI, 70.7, 82.1), and for those with psychiatric comorbidity, it was 86.9 (95% CI, 69.2, 109.1). The rate of incident opioid use did not differ between persons with UC with and without psychiatric comorbidity (RR 1.14; 95% CI, 0.90, 1.45). The RR was higher when comparing persons with UC and no psychiatric comorbidity with a matched reference cohort with no psychiatric comorbidity (1.78; 95% CI, 1.64, 1.93) than when comparing opioid use in persons with UC and psychiatric comorbidity to matched reference cohort with psychiatric comorbidity (RR of incident opioid use was 1.51 [95% CI, 1.17, 1.96]). The adjusted RR was lower for opioid incidence, but it was still greater when comparing persons with UC and their reference cohort without psychiatric comorbidity (1.34; 95% CI, 1.22, 1.47) versus those with psychiatric comorbidity (1.19; 95% CI, 0.92, 1.56).

### Prevalence of Opioid Use

On average over the study period, the IBD cohort had twice the age- and sex-standardized prevalence per 1000 persons of opioid use (270.58; 95% CI, 266.50, 276.41) as the reference cohort (129.33 [95% CI, 127.90, 130.83]; RR 2.09 [95% CI, 2.05, 2.14]). The prevalence ratio (PR) was similar across the 18-44 (PR 2.11; 95% CI, 2.06, 2.17) and 45-64 years groups (PR 2.16; 95% CI, 2.10, 2.23). However, it was lower in those 65+ years (PR 1.97; 95% CI, 1.88, 2.07, *P* = .006 for interaction). The PR was higher among females with IBD compared to their matched reference cohort (PR 2.22; 95% CI, 2.17, 2.27) than among males with IBD and their matched reference cohort (PR 1.94; 95% CI, 1.88, 1.99).

The average age- and sex-standardized prevalence of opioid use per 1000 persons over the entire study period among persons with IBD and no mood disorder was 244.11 (95% CI, 239.16, 249.17); this prevalence was much higher at 438.45 (95% CI, 421.03, 456.59) among persons with psychiatric comorbidity (Supplementary Table S6). However, the IBD cohort versus reference cohort adjusted PR was higher among persons without psychiatric comorbidity (2.09; 95% CI, 2.01, 2.11) compared to those with psychiatric comorbidity (1.77; 95% CI, 1.69, 1.86).

The differences between incident and prevalent opioid use on multivariate analysis was that in the prevalent opiate use analysis older age and longer disease duration were associated with higher opiate use than reference controls. Sex did not predict prevalent opioid use (Supplementary Table S7).

### Patterns of Opioid Use

**Figure 1.**
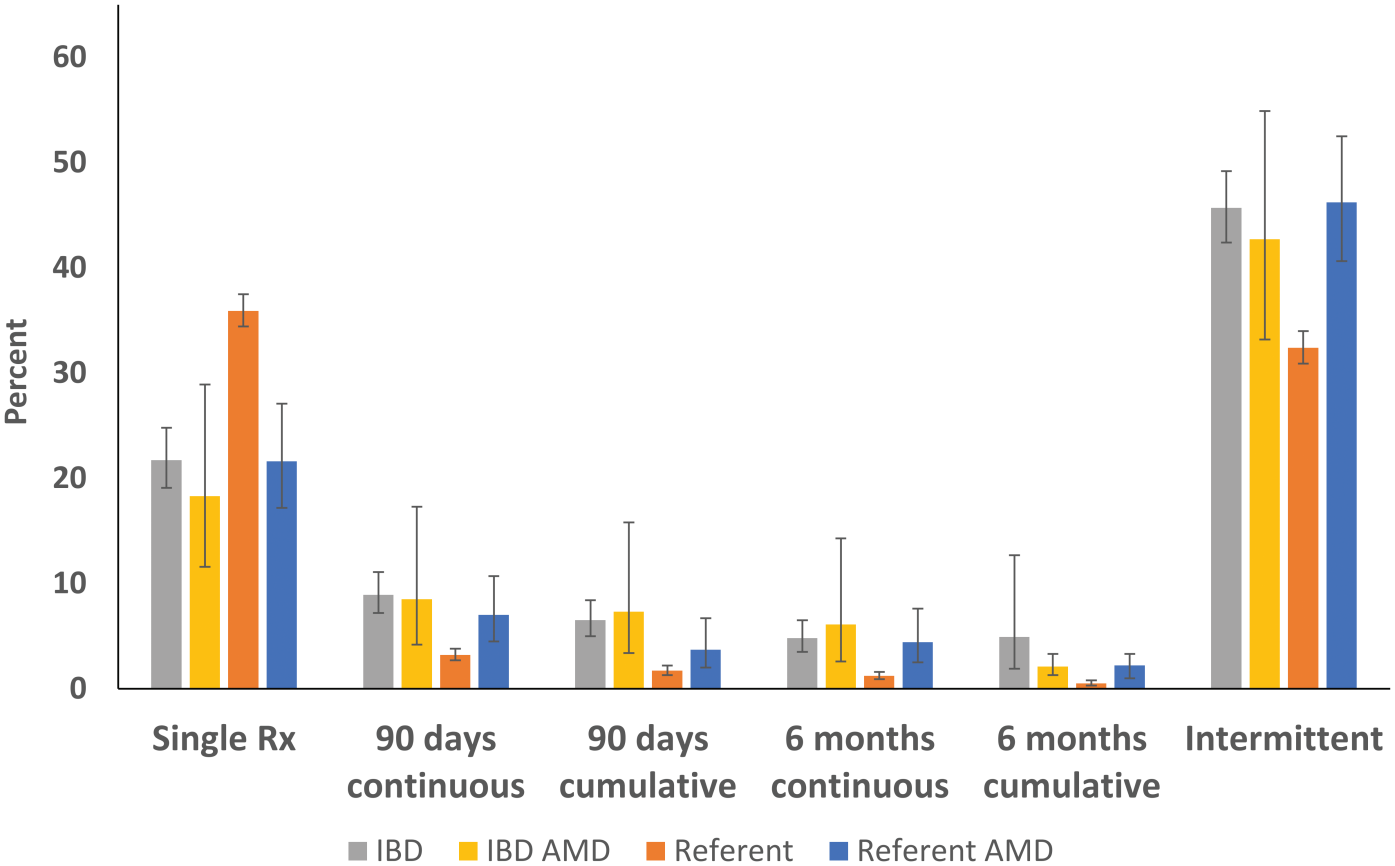
Rates of discontinuation of opioid use with 30 days use, within 90 days, within 6 months, and for cumulative opioid use over 90 days in persons with IBD and the reference cohort with and without anxiety and mood disorders (AMD).

Among persons with IBD, psychiatric comorbidity was not associated with their rates of discontinuation of opioid use within 30 days of use, within 90 days or within 6 months, or in their cumulative opioid use over 90 days (Figure 1). Among persons with no psychiatric comorbidity, persons with IBD were less likely to discontinue opioids at 30 days (RR = 0.85; 95% CI, 0.82, 0.88); they were also 2-3 times more likely to use opioids either continuously or cumulatively over 90 days (RR = 2.42; 95% CI, 1.95, 3.01), and 6 months compared to the reference cohort (RR = 3.35; 95% CI, 2.40, 4.67). In the reference cohort, those with psychiatric comorbidity were significantly less likely to discontinue opioids within 30 days (RR = 0.91; 95% CI, 0.87, 0.95), and 2-3 times more likely to continue over 90 days (2.09; 95% CI, 1.49, 2.94) or 6 months (RR = 3.19; 95% CI, 1.96, 5.17) or cumulatively over 90 days (RR = 2.13; 95% CI, 1.32, 3.45) compared with the reference cohort without psychiatric comorbidity. Similar results for the timing of discontinuation were evident for prevalent cases compared to the reference cohort in those with and without psychiatric comorbidity (Supplementary Table S8).

## Discussion

Using population-based data spanning more than 20 years, we compared the incidence, prevalence, and patterns of opioid use in adults with IBD compared to adults without IBD. Over the study period, over 1 in 4 persons with IBD had at least 1 opioid dispensation. The highest rate of opioid dispensation was among persons ages 18-44 years. People with IBD were twice as likely to be prescribed opioids compared with the reference cohort across the age spectrum. Older age, being female, longer disease duration, and later year of diagnosis were all associated with lower rates of opioid use. Being in the poorest socioeconomic quintile and having greater healthcare needs or utilization were all associated with higher rates of opioid use. Psychiatric comorbidity was associated with increased incidence rates of opioid use in the IBD and reference cohorts, but the magnitude of the effect was lower in the IBD cohort. Psychiatric comorbidity was not associated with patterns of discontinuation of opioids in the IBD cohort in contrast to the reference cohort. These findings suggest that IBD may be the more relevant factor to understand higher opioid rates than psychiatric comorbidity. A similar pattern of opioid use was evident in CD and in UC. However, the incidence of opioid use was higher in CD than UC.

In a systematic review and meta-analysis of 31 mostly US-based studies the prevalence of opioid use in IBD in the outpatient setting was 21% (95% CI, 13%-30%).^24^ Our findings fall within the upper limit of this range. However, these studies had heterogeneous definitions of opioid use, ranging from any prescription in the past 2 years to monthly prescriptions over 3 months, making it challenging to meaningfully interpret the meta-analysis. In a subgroup analysis of five studies of opioid users of >90 days in 6 months or >3 times weekly, the prevalence was 13% (95% CI, 6%-24%). Physicians providing care for IBD patients need to be vigilant with their prescribing of opioids whether it is for pain or diarrhea control, as opioid use is associated with excess mortality in the IBD population.^12^ While there are evident risks of opioid medication, the long-term benefits of chronic opioid use are uncertain.^25,26^ Persistent opioid use rises even after short-term use, to as high as 43% after 31 days of use.^27^ In a national US insurance claims database, from 2009 to 2015, 21% of adults with IBD were prescribed opioids.^28^ Persistent use was evident in 35% of those who were newly prescribed opioids, whether or not, there was a recent surgery.

It is unclear why the association of psychiatric comorbidity with opioid use was relatively lower in persons with IBD than the reference cohort. This may reflect a ceiling effect of opioid use due to IBD, but it may be a caution on the part of the prescriber. If the individual is already being managed medically for the IBD, and is presenting with significant mental distress, there may be a reluctance to prescribe an additional medication which requires careful oversight and has a risk of addiction. There are other complex provider–patient interaction factors that could ultimately result in less opioid dispensations in persons with IBD.

We identified several factors associated with incident opioid use. We observed a female predominance in age-standardized analyses of incident use, but this changed after we accounted for disease severity, comorbidity, medication use, healthcare visits, and socioeconomic status. After multivariable analysis, we observed that females were non-significantly more likely to have prevalent opioid use (PR 1.03; 95% CI, 0.99-1.06). Prior studies have reported that being female, substance abuse, depression, somatiform symptom reporting, antidepressant use, prior gastrointestinal surgery, biologic use, and corticosteroid use are associated with prevalent opioid use.^29,30^ Our findings regarding psychiatric comorbidity are consistent with these observations. The combination of our findings regarding sex with those of prior studies suggests that males may be at higher risk of initiating opioid therapy after accounting for confounders, but that females may be more likely to use opioids chronically. Differences in measured confounders as well as unmeasured confounders may also play a role in differences across studies. Similarly, we found that younger age was associated with an increased incidence of opioid use, but lower prevalence of use, suggesting older age is associated with chronicity of opioid use. In a population-based study from Denmark from 1996 to 2021, older age of IBD onset was associated with higher rates of opioid use in the first year after diagnosis; however, this study had a different design and question than ours.^31^

Our study has limitations. We could not assess the use of opioids that are available over the counter, such as acetaminophen–codeine combinations. Importantly, we did not know the indication for which opioids were prescribed; they could have been for non-IBD-related pain syndromes. Since the study relied on administrative data to identify psychiatric comorbidity, individuals who do not seek care for mental health or who seek care from nonphysician providers will not have been captured in our datasets, thus we could have underestimated the number of individuals affected by psychiatric comorbidity in persons with IBD and in the reference cohort, alike. Nonphysician providers who may not have been captured included some nurse practitioners who are salaried and psychologists who are either salaried or work privately for which their services are not reimbursed by the provincial healthcare systems. Further, the data on opioid use are fundamentally prescription data, which indicate what was dispensed but we cannot affirm what was actually used. Since persons with IBD have more physician visits than persons in the reference cohort, there could be a risk for surveillance bias; however, we specifically adjusted for annual physician visits and number of prescription drug classes used to account for this potential bias. Finally, we did not assess whether opioid use may affect the risk of incident psychiatric disorders and this should be explored in future studies. Despite these limitations, a major strength of the study is the use of population-based data over a 20-year period, with comprehensive physician visit and prescription medication capture. These findings warrant replication in other jurisdictions.

Our study reaffirms the higher rate of opioid use in persons with IBD compared to the general population and that this impacts all demographics of persons with IBD. Nearly 1 in 20 people with IBD will continue opioids for at least 6 months consecutively. Psychiatric comorbidity, which is more highly prevalent in IBD, was associated with greater relative opioid use in persons without IBD than with IBD but did not influence discontinuation of opioids. Comorbidities, lower socioeconomic status, and male sex were also associated with incident opioid use. The general harm of opioid use is well recognized. Clinicians need to be careful when considering initiation of opioid therapy in the context of psychiatric comorbidity and to review existing opioid use in their patients with IBD and work to reduce or eliminate use when appropriate.

## CIHR Team

Members of the CIHR Team in Defining the Burden and Managing the Effects of Psychiatric Comorbidity in Chronic Immunoinflammatory Disease are: R.A.M., J.M.B., J.S., S.B.P., A.S., L.M.L., C.A.H., R.E.-G., PhD, A.K., J.D.F., C.N.B., L.A.G., Lindsay Berrigan, Ryan Zarychanski, Christine Peschken, James Marriott, Kaarina Kowalec.

izae188_suppl_Supplementary_Material

## Data Availability

The datasets presented in this article are not readily available because they belong to Manitoba Health and all data relevant to the article are presented within the article. **Guarantor of the article:** C.N.B. is the article guarantor.
