# Supplementary material for: Psychiatric Comorbidity Does Not Enhance Prescription Opioid Use in Inflammatory Bowel Disease as It Does in the General Population
Source: Inflamm Bowel Dis. 2024 Sep 3;31(2):386–93. doi: 10.1093/ibd/izae188 (PMC11808568; doi:10.1093/ibd/izae188)
Supplement: izae188_suppl_Supplementary_Material [file izae188_suppl_supplementary_material.zip › Supplementary Figure legend.docx]

**Supplementary Figure 1**

The average age-standardized incidence rate per 1000 person-years by age cohort
