# Supplementary material for: Psychiatric Comorbidity Does Not Enhance Prescription Opioid Use in Inflammatory Bowel Disease as It Does in the General Population
Source: Inflamm Bowel Dis. 2024 Sep 3;31(2):386–93. doi: 10.1093/ibd/izae188 (PMC11808568; doi:10.1093/ibd/izae188)
Supplement: izae188_suppl_Supplementary_Material [file izae188_suppl_supplementary_material.zip › Opioids in IBD July 2024 supplementary tables clean.docx]

Supplemental Table 1. Case definitions for psychiatric comorbidity in administrative health data

| Condition | ICD-9-CM/ICD-10-CA |
| --- | --- |
| Depression | 296.2, 296.3, 298.0, 300.4, 311/ F32, F33, F34 |
| Anxiety | 300.0/F41 |
| Bipolar disorder | 296.0, 296.1, 296.04, 296.14, 296.4, 296.44, 296.5, 296.44, 296.5, 296.54, 296.6, 296.7, 296.8/ F31 |

Supplemental Table 2 Reason for truncation of follow up to March 31, 2018. Referent cohort participants with cancer or palliative care were dropped prior to their matching

| Truncation reason | Reference cohort | IBD cohort |
| --- | --- | --- |
| Cancer |  | 778 (14.9%) |
| Death | 1218 (4.7%) | 193 (3.7%) |
| End of Study | 21282 (81.4%) | 2795 (72.%) |
| Moved | 3650 (14.0%) | 454 (8.7%) |
| Palliative care |  | 13 (0.2%) |

Supplemental Table 3. Likelihood of receiving tricyclic antidepressants, selective serotonin reuptake inhibitors (SSRI), selective serotonin and norepinephrine reuptake inhibitors (SNRI) or non-steroidal anti-inflammatory drugs (NSAID) prescription in the year before the initial opioid dispensation

|  | IBD (n=1420) | Controls (n=5693) | RR 95% CI |
| --- | --- | --- | --- |
| Tricyclic antidepressant | 55 (3.9%) | 130 (2.3%) | 1.60 (1.24, 2.31) |
| SSRI | 101(7.1%) | 330 (5.8%) | 1.23 (0.99, 1.52) |
| NSRI | 30 (2.1%) | 97 (1.7%) | 1.24 (0.83, 1.86) |
| NSAID | 197 (13.9%) | 904 (15.9%) | 0.87 (0.76, 1.01) |

| Supplemental Table 4. Factors associated with incident opioid use (incidence rate ratio, 95% confidence interval, CI) Effect estimates for adjustment covariates for the incident CD cohort |
| --- |

| **Label** | **Effect estimate** | **95% CI** |
| --- | --- | --- |
| Age 45-64 vs 18-44 | 0.79 | ( 0.73, 0.86) |
| Age 65+ vs 18-44 | 0.70 | ( 0.62, 0.79) |
| Female vs male | 0.95 | ( 0.88, 1.02) |
| Urban vs rural | 0.98 | ( 0.91, 1.05) |
| ADG 1 vs 0 | 1.10 | ( 0.99, 1.21) |
| ADG 2+ vs 0 | 1.65 | ( 1.29, 2.12) |
| ATC4 drug classes 2-3 vs 0-1 | 1.23 | ( 1.13, 1.35) |
| ATC4 drug classes 4+ vs 0-1 | 1.38 | ( 1.23, 1.53) |
| Prior CD-specific procedure | 0.76 | ( 0.57, 1.03) |
| Prior physician visits: 4-7 vs 0-3 | 1.23 | ( 1.12, 1.35) |
| Prior physician visits: 8 or more vs 0-3 | 1.21 | ( 1.08, 1.36) |
| SEFI: Poorest vs Richest quartile | 1.14 | ( 1.03, 1.27) |
| IMID disease duration among cases (1 extra year) | 0.83 | ( 0.81, 0.86) |
| IMID diagnosis year | 0.93 | ( 0.93, 0.94) |
| CASE*PSYCH interaction term | 0.86 | ( 0.62, 1.18) |

Legend: ADG: John Hopkins Adjusted Clinical Group System Aggregated Diagnosis Groups, ATC4: Anatomical Therapeutic Chemical (ATC) Classification System 4, SEFI: Socioeconomic Factor Index, CASE*PSYCH interaction term: IBD case vs control with and without anxiety and/or mood disorders

Supplemental Table 5. Factors associated with incident opioid use (incidence rate ratio, 95% confidence interval, CI) Effect estimates for adjustment covariates for the incident UC cohort

| **Label** | **Effect estimate** | **95% CI** |
| --- | --- | --- |
| Age 45-64 vs 18-44 | 0.80 | ( 0.75, 0.86) |
| Age 65+ vs 18-44 | 0.69 | ( 0.62, 0.76) |
| Female vs male | 0.88 | ( 0.83, 0.94) |
| Urban vs rural | 1.01 | ( 0.94, 1.08) |
| ADG 1 vs 0 | 1.22 | ( 1.12, 1.33) |
| ADG 2+ vs 0 | 1.15 | ( 0.92, 1.45) |
| ATC4 drug classes 2-3 vs 0-1 | 1.17 | ( 1.07, 1.27) |
| ATC4 drug classes 4+ vs 0-1 | 1.25 | ( 1.13, 1.38) |
| Prior UC-specific procedure | 1.48 | ( 1.10, 1.98) |
| Prior physician visits: 4-7 vs 0-3 | 1.35 | ( 1.24, 1.47) |
| Prior physician visits: 8 or more vs 0-3 | 1.50 | ( 1.35, 1.67) |
| SEFI: Poorest vs Richest quartile | 1.07 | ( 0.96, 1.19) |
| IMID disease duration among cases (1 extra year) | 0.91 | ( 0.90, 0.93) |
| IMID diagnosis year | 0.94 | ( 0.93, 0.94) |
| CASE*PSYCH interaction term | 0.89 | ( 0.68, 1.17) |

Legend: ADG: John Hopkins Adjusted Clinical Group System Aggregated Diagnosis Groups, ATC4: Anatomical Therapeutic Chemical (ATC) Classification System 4, SEFI: Socioeconomic Factor Index, CASE*PSYCH interaction term: IBD case vs control with and without anxiety and/or mood disorders

| Supplemental Table 6. Opioid Prevalence per 1,000, 1997-2017, Age/Sex Standardized to 2010 Canadian population, by psychiatric comorbidity (PC)   \|  \| PC \| Observed events \| Population time \| Standardized rate (upper, lower limits) \| Case/control adjusted prevalence ratio \| 95% CI \| \| --- \| --- \| --- \| --- \| --- \| --- \| --- \| \| IBD Case \| No \| 9418 \| 38818 \| 244.11 \| 2.06 \| 2.01, 2.11 \| \| IBD Case \| Yes \| 2747 \| 6019 \| 438.45 \| 1.77 \| 1.69, 1.86 \| \| Reference cohort \| No \| 25331 \| 214842 \| 118.37 \|  \|  \| \| Reference cohort \| Yes \| 5125 \| 20648 \| 247.38 \|  \|  \| |
| --- | --- | --- | --- | --- | --- | --- | --- | --- | --- | --- | --- | --- | --- | --- | --- | --- | --- | --- | --- | --- | --- | --- | --- | --- | --- | --- | --- | --- | --- | --- | --- | --- | --- | --- | --- |

PC refers to mood and/or anxiety disorder or bipolar disorder

Supplemental Table 7. Patterns of opioid use over 30 days, 90 days and 6 months for IBD and controls with and without Anxiety and/or Mood Disorders (AMD) in prevalent cases. Relative rates (RR) are presented for IBD cases with and without AMD, controls wit and without AMD and for IBD versus controls with and without AMD

| Discontinuing within 30 d | Case=1  Psych=1 | Case=1  Psych=0 | Control=1  Psych=1 | Control=1  Psych=0 |
| --- | --- | --- | --- | --- |
|  | 78% | 75.5% | 80.4% | 88.7% |
| Case/psych  Case/no psych RR | 1.03  (0.94, 1.14) |  |  |  |
| Control/psych  Control/no psych RR |  |  | 0.91  (0.87, 0.95) |  |
| Case /Psych  Control/psych  RR | 0.84  (0.62, 1.13) |  |  |  |
| Case/No psych  Control/No psych |  | 0.68  (0.63, 0.74) |  |  |
| Case*Psych;p=0.02 |  |  |  |  |
|  |  |  |  |  |
| Proportion Continuous 90 days Opioid use | 7.9% | 9.4% | 8.1% | 3.9% |
| Case/psych  Case/No psych RR | 0.84  (0.45, 1.56) |  |  |  |
| Control/psych  Control/no psych RR |  |  | 2.09  (1.49, 2.94) |  |
| Case/psych  Control/psych | 0.97  (0.50, 1.91) |  |  |  |
| Case/No psych  Control/no psych |  | 2.42  (1.95, 3.01) |  |  |
| Case*psych;p=0.012 |  |  |  |  |
|  |  |  |  |  |
| Proportion Continuous 6 months Opioid use | 4.7% | 4.7% | 4.5% | 1.4% |
| Case/psych  Case/No psych RR | 1.0  (0.44, 2.27) |  |  |  |
| Control/psych  Control/no psych RR |  |  | 3.19  (1.96, 5.17) |  |
| Case/psych  Control/psych | 1.05  (0.43, 2.56) |  |  |  |
| Case/No psych  Control/no psych |  | 3.35  (2.40, 4.67) |  |  |
| Case*psych;p=0.017 |  |  |  |  |
| Proportion Cumulative 90 days Opioid use | 6.3% | 6.2% | 4.3% | 2.0% |
| Case/psych  Case/No psych RR | 1.02  (0.50, 2.06) |  |  |  |
| Control/psych  Control/no psych RR |  |  | 2.13  (1.32, 3.45) |  |
| Case/psych  Control/psych | 1.48  (0.66, 3.29) |  |  |  |
| Case/No psych  Control/no psych |  | 3.09  (2.33, 4.11) |  |  |
| Case*psych;p=0.088 |  |  |  |  |

Supplemental Table 8. Factors associated with prevalent opioid use (prevalence ratio, 95% confidence interval, CI)

| Label | Effect estimate | 95% CI |
| --- | --- | --- |
| Age 65+ vs 18-64 | 1.050 | (1.010, 1.092) |
| Female vs male | 1.026 | (0.994, 1.059) |
| Urban vs rural | 0.992 | (0.959, 1.026) |
| ADG 1 vs 0 | 1.099 | (1.075, 1.124) |
| ADG 2+ vs 0 | 1.206 | (1.159, 1.256) |
| ATC4 drug classes 2-3 vs 0-1 | 1.214 | (1.179, 1.250) |
| ATC4 drug classes 4+ vs 0-1 | 1.507 | (1.459, 1.557) |
| Prior IBD-specific procedure | 1.058 | (0.982, 1.140) |
| Prior physician visits: 4-7 vs 0-3 | 1.256 | (1.223, 1.290) |
| Prior physician visits: 8 or more vs 0-3 | 1.513 | (1.466, 1.562) |
| SEFI: Poorest vs Richest quartile | 1.505 | (1.435, 1.577) |
| IMID diagnosis year | 1.000 | (0.997, 1.003) |
| IMID disease duration among cases (1 extra year) | 1.006 | (1.002, 1.011) |
| CASE*PSYCH interaction | 0.999 | (0.941, 1.061) |

Legend: ADG: John Hopkins Adjusted Clinical Group System Aggregated Diagnosis Groups, ATC4: Anatomical Therapeutic Chemical (ATC) Classification System 4, SEFI: Socioeconomic Factor Index, CASE*PSYCH interaction term: IBD case vs control with and without anxiety and/or mood disorders
