# Supplementary figures and images for: Psychiatric Comorbidity Does Not Enhance Prescription Opioid Use in Inflammatory Bowel Disease as It Does in the General Population
Source: Inflamm Bowel Dis. 2024 Sep 3;31(2):386–93. doi: 10.1093/ibd/izae188 (PMC11808568; doi:10.1093/ibd/izae188)

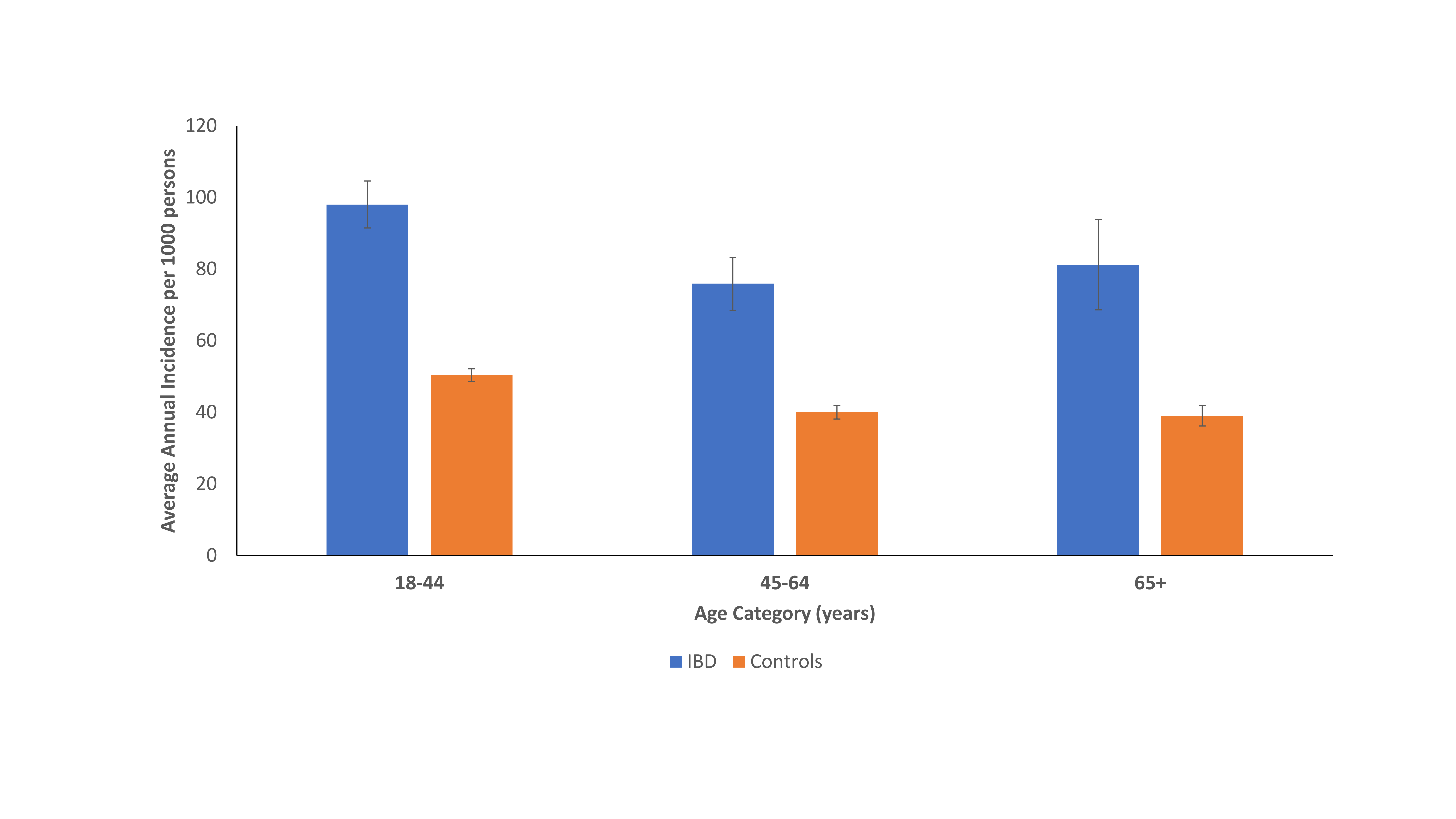

Supplement: izae188_suppl_Supplementary_Material [file izae188_suppl_supplementary_material.zip › SuppFig1_06.15.24_opiates and IBD.tif]
